# Research progress on high-concentration oxygen therapy after cerebral hemorrhage

**DOI:** 10.3389/fneur.2024.1410525

**Published:** 2024-07-29

**Authors:** He Zeng, Dakai Zeng, Xiaoping Yin, Wumiao Zhang, Moxin Wu, Zhiying Chen

**Affiliations:** ^1^Department of Neurology, Clinical Medical School of Jiujiang University, Jiujiang, Jiangxi, China; ^2^Jiujiang Clinical Precision Medicine Research Center, Jiujiang, Jiangxi, China; ^3^Department of Anorectal Surgery, Third Affiliated Hospital of Wenzhou Medical University, Zhejiang, China

**Keywords:** cerebral hemorrhage, secondary ischemia, oxygen metabolic rate, hyperbaric oxygen, normobaric high-concentration oxygen

## Abstract

Recently, the role of high-concentration oxygen therapy in cerebral hemorrhage has been extensively discussed. This review describes the research progress in high-concentration oxygen therapy after cerebral hemorrhage. High-concentration oxygen therapy can be classified into two treatment methods: hyperbaric and normobaric high-concentration oxygen therapy. Several studies have reported that high-concentration oxygen therapy uses the pathological mechanisms of secondary ischemia and hypoxia after cerebral hemorrhage as an entry point to improve cerebral oxygenation, metabolic rate, cerebral edema, intracranial pressure, and oxidative stress. We also elucidate the mechanisms by which molecules such as Hypoxia-inducible factor 1-alpha (HIF-1α), vascular endothelial growth factor, and erythropoietin (EPO) may play a role in oxygen therapy. Although people are concerned about the toxicity of hyperoxia, combined with relevant literature, the evidence discussed in this article suggests that as long as the duration, concentration, pressure, and treatment interval of patients with cerebral hemorrhage are properly understood and oxygen is administered within the treatment window, it can be effective to avoid hyperoxic oxygen toxicity. Combined with the latest research, we believe that high-concentration oxygen therapy plays an important positive role in injuries and outcomes after cerebral hemorrhage, and we recommend expanding the use of normal-pressure high-concentration oxygen therapy for cerebral hemorrhage.

## 1 Introduction

Spontaneous intracerebral hemorrhage (ICH) refers to the hemorrhage of brain parenchyma caused by vascular rupture caused by non-traumatic causes. With an increase in population age and widespread use of antithrombotic drugs, risk factors such as hypertension, diabetes, obesity, and alcohol abuse have increased, and the incidence of ICH is also increasing (1). Its high mortality and disability rates are closely related to neuronal damage caused by pathological reactions such as perifocal hypoxia after ICH. Surviving patients often experience permanent sequelae (2).

Brain injury after ICH can be classified into primary and secondary injuries. The primary injury is mechanical compression and expansion of the hematoma, which are key factors in determining the progression and outcome of ICH. They are generally caused by continued bleeding from ruptured blood vessels (3), usually occurring within 6 h after ICH, which can induce a space-occupying effect, compress blood vessels, reduce the volume of the vascular bed, increase intracranial pressure (ICP), decrease local perfusion, inhibit membrane ion pump activity (4), increase intracellular sodium ions, decrease intracellular crystal osmotic pressure, and form cytotoxic edema (5). The decrease of calcium ions in serum impairs the thrombin cascade reaction, and coagulation dysfunction (3) forms thrombosis, resulting in cerebral microcirculation obstruction and decreased oxygenation.

**Figure 1:**
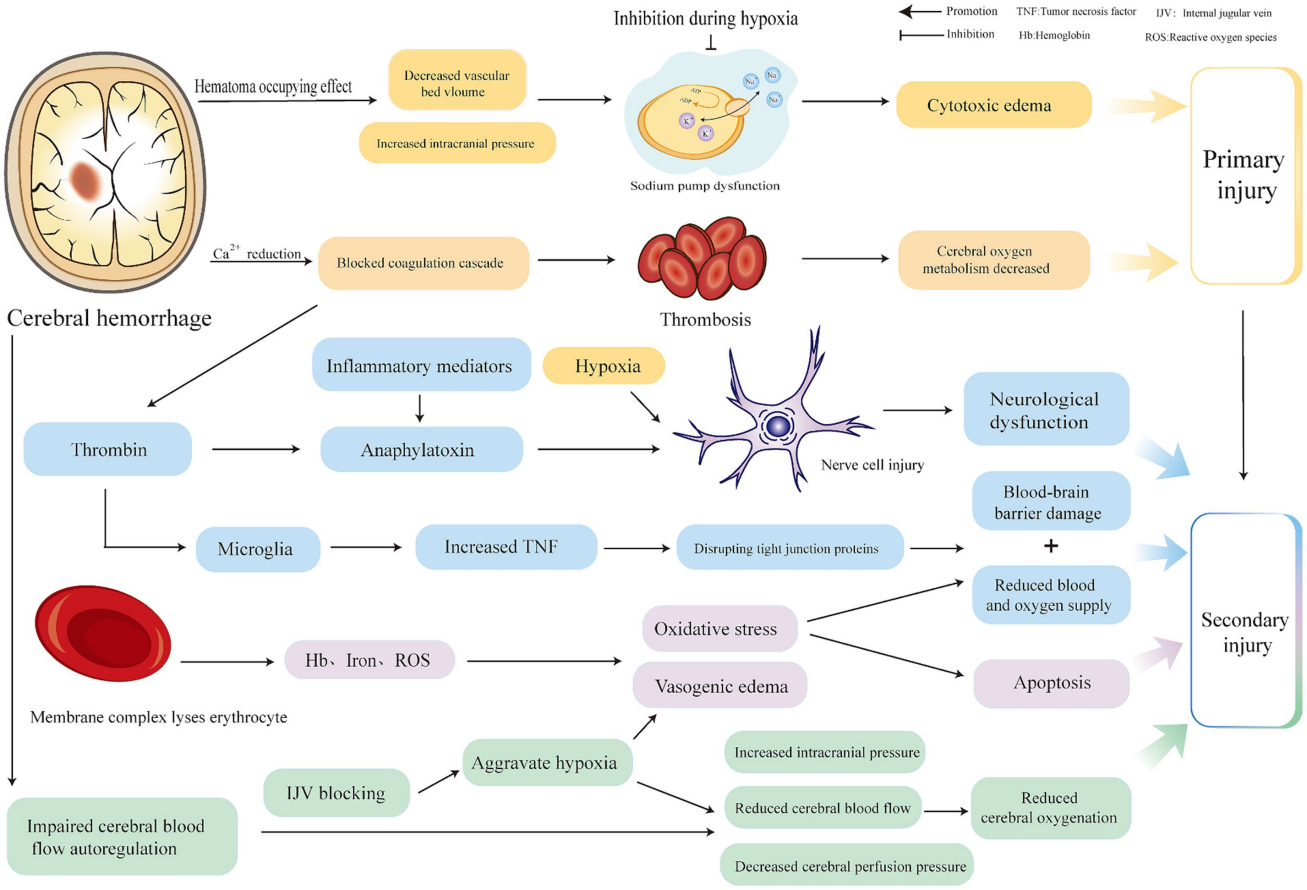
Injury mechanism of cerebral hemorrhage.

Hematoma components can induce secondary ICH. Thrombin is one of the components of hematoma. A high concentration of thrombin activated by the thrombin cascade reaction after ICH is closely associated with secondary ICH injury (4, 6). Thrombin can induce the production of inflammatory mediators, cause nerve cell damage (7), and influence neurological outcomes after ICH. Thrombin can also cleave the protease-activated receptors (PARs) receptor in microglia, which in turn phosphorylates the Src family kinase, thereby aggravating brain edema and the destruction of the blood–brain barrier. Stimulated microglia can also destroy tight junction proteins by up-regulating the expression of tumor necrosis factor (TNF) (6), increasing blood–brain barrier permeability. The complement cascade activated by thrombin may promote inflammation and edema after ICH through anaphylactoid toxins, or it may lyse red blood cells through membrane complexes to produce hemoglobin, iron, and oxygen free radicals, promote edema and oxidative stress and accelerate apoptosis after ICH, death, and blood–brain barrier disruption (4, 6). Red blood cells are also one of the components of hematoma. After intracerebral hemorrhage, the blood overflows from the ruptured blood vessels, and some red blood cells that are not completely phagocytized by microglia and infiltrating macrophages are directly released into the central system. Substances such as hemoglobin, iron, and carbonic anhydrase-1 are neurotoxic and are closely related to secondary brain injury after ICH (8). In addition, the automatic adjustment function of cerebral blood flow is based on a formula for cerebral blood flow, cerebral perfusion pressure (CPP), and cerebral vascular resistance (CVR). CVR can be adjusted to ensure the supply of cerebral blood flow (CBF) when ICP increases and CPP decreases. Expanded hematoma and high intracranial pressure after ICH cause decompensation of this regulation, further reducing CPP and aggravating cerebral ischemia (9). It is important to note that because hypertension is the most common cause of ICH (10), antihypertensive measures are often taken to alleviate the expansion of ICH hematoma; however, this is undoubtedly a challenge to the automatic regulation of cerebral blood flow. Therefore, attention should be paid to the tolerance of patients with hypertensive ICH to rapid blood pressure reduction; otherwise, insufficient cerebral perfusion will occur (11). Adnan et al. recommended that for intensive blood pressure reduction in ICH, systolic blood pressure should be controlled at 130–150 mmHg (12). Notably, cerebral venous outflow disorder also has a significant impact on the pathological mechanisms of ICH. Some researchers have found that cerebral venous outflow disorders are closely related to cerebrospinal fluid dynamics (13), especially in the internal jugular vein (IJV), which is the main route of brain drainage (14). Feng et al. (14) found that among patients with ICH, those with positive internal jugular venous reflux had larger perihematomal edema (PHE) volume than those with negative internal jugular venous reflux and demonstrated a positive correlation between jugular venous reflux (JVR) and PHE volume. When JVR worsens, it can further increase ICP, reduce CBF and CPP, aggravate vasogenic edema, and reduce cerebral oxygenation (14, 15). In general, secondary injuries can include vasogenic edema, neuroinflammatory reactions, blood–brain barrier damage, decompensation of cerebral blood flow autoregulation, excessive lowering of blood pressure, and cerebral venous return disorder, which are related. The pathological mechanisms are different, but to a certain extent, they can synergistically aggravate pathological reactions such as brain edema, neuroinflammatory reactions, high ICP, low CPP, oxidative stress, cell apoptosis, and destruction of the blood–brain barrier after ICH. From a comprehensive literature review, we believe that ischemia and hypoxia after ICH intersect and interact with these pathological reactions (16, 17). (The details are shown in Figure 1.)

A green stroke channel has been established clinically, and the slogan “time is brain” has been put forward because ICH is dangerous and progresses rapidly. Although there is currently no specific clinical treatment for ICH, the pathological basis of ICH can be explored to improve patient prognosis. Recently, high-concentration oxygen therapy has become increasingly active in the public eye. This emerging and effective intervention can alleviate ischemic hypoxic conditions after ICH, reduce intracranial pressure and cerebral edema, and improve neuroinflammatory reactions and other adverse effects. This study aimed to explore the progress in the efficacy of high-concentration oxygen therapy for ICH. Clinically, common high-concentration oxygen therapies used after ICH can be categorized into hyperbaric oxygen (HBO) and normobaric oxygen (NBO).

## 2 Hyperbaric oxygen therapy

HBO is a treatment method in which a patient intermittently inhales 100% pure oxygen in a high-pressure chamber at a pressure greater than sea level [>1 Atmospheres Absolute (ATA)] (9). Henry’s law provides an understanding of the basis of HBO treatment; that is, the proportion of dissolved oxygen is positively related to the partial pressure of oxygen (18). HBO can increase the oxygen partial pressure in the blood and damaged tissues by increasing the dissolved oxygen content, improving cerebral oxygenation, and increasing the oxygen metabolic rate (19, 20) (Table 1).

**Table 1:** The possible mechanism and effect of high-concentration oxygen therapy after ICH and the potential therapeutic effect of high-concentration oxygen therapy.

Therapies	Experimental Subject	Action Pathway	Target	Direction of application	Experimental scheme	Experimental result	Effects	References
HBO	health persons	NO/O2-/ONOO-	Endothelial cells and vascular smooth muscle cells	Upregulation	Breathing was performed at 2.5 ATA for 60 min, and the air was interrupted twice for 5 min. The peripheral blood flow of some organs was compared before and after HBO exposure for 10 min.	The blood flow of some organs continued to rise within 10 min after HBO.	Increase dissolved oxygen, increase organ blood flow.	(21)	
	Diabetic patients with ICH	/	Neuron	/	The neurological scores of the two groups of diabetic stroke patients were compared within 6 months after receiving 2.5ATA HBO and 1.5ATA 100% NBO once a day for 60 min and 30 consecutive days.	Within 6 months of follow-up, the neurobehavioral scores of the two groups were improved, but the effect of 2.5ATAHBO group was more significant.	High concentration of oxygen can reduce blood glucose levels and improve NIHSS.	(22)	
	ICH Rats	HIF/VEGF	Neuron	Upregulation	/	/	Improve brain microcirculation, promote angiogenesis, repair damaged neurons.	(23)	
	ICH Rats	MMPs/occludin	Neuron	Downregulation	After ICH induction, mice were exposed to air, NBO, and 3ATA HBO for 60 min. The occludin, MMPs, and HIF-1α at 30 min, 60 min, and 120 min after treatment were compared among the three groups.	3ATA HBO treatment for 30 min had the most significant effect on preventing the degradation of occludin, activating MMP-9 and reducing HIF-1α expression.	Inhibition of MMP-mediated proteolysis of blood–brain barrier components and hif-1a-induced upregulation of VEGF promotes occludin to protect BBB and reduce hemorrhagic edema.	(24)	
	ICH Rats	HIF/VEGF	Endothelial cell	Downregulation (early phase)				(24)	
	ICH Rats	proMMP-9/occludin	Neuron	Downregulation				(24)	
	ICH Rats	TNF-α	Microglia	Downregulation	The rats were randomly divided into three groups sham operation group (sham operation + HBOP treatment for 5 days, 100% oxygen 2 ATA, 60 min per day), ICH group (normal pressure air 5 days before ICH), HBOP group (HBOP treatment 5 days before ICH). The number of microglia in the three groups at 12 h, 24 h and 72 h after the experiment was 100% compared.	Compared with the ICH group, the expression of microglia in the HBOP group was significantly reduced at 12 h, 24 h and 72 h.	HBOP can reduce neuronal degeneration by reducing the expression of microglia.	(25)	
	MACO Rats	Glucose metabolic	ischemic tissue	Upregulation	HBO (3 ATA for 1 h with 100% oxygen)was started 1 h after MACO in rats. Local cerebral glucose utilization was measured before ischemia, 1 h and 3 h after ischemia, and neurological deficits and infarct volume were evaluated 24 h after ischemia.	Ischemia was improved in the HBO group 1 h after MCAO. The cortical infarct volume at 24 h after ischemia was negatively correlated with the percentage of low glucose metabolism in the ischemic core at 3 h after ischemia.	HBO increases the rate of glucose metabolism in the brain, improves the degree of ischemia and reduces the infarct size.	(26)	
	Critical limb ischemic rats (CLI)	HIF/VEGF	Endothelial progenitor cells	Upregulation	Forty SD rats were randomly divided into sham operation control (SC), CLI, CLI + HBO [5 days of intermittent 2.4ATA HBO therapy/3 h per day after CLI], CLI + EPC and CLI + HBO + EPC. Their levels of endothelial progenitor cells *in vivo* were compared on day 4 and day 15 after surgery.	At 5 and 14 days after operation, the level of cell circulation in the endothelial group of CLI-HBO-EPC was the highest.	HBO can promote the regeneration of endothelial progenitor cells and increase the blood flow in the ischemic area.	(27)	
	Rats with focal cerebral ischemia	HIF/EPO	Neuron	Upregulation	The treated rats were randomly divided into control group and HBO (100% HBO 2 ATA of 1-h duration once every other day for five sessions) group. The cerebral infarction volume, HIF-1α and EPO levels were compared between the two groups at 4 h, 8 h and 24 h after treatment.	At 4 h after the beginning of the experiment, HIF-1α and EPO in the HBO group increased, and the volume of cerebral infarction decreased.	HBO can up-regulate HIF-1α and its target gene EPO, prevent ischemic neuronal changes and reduce apoptosis.	(28)
**NBO**	ICH Rats	MMPs/occludin	Neuron	Downregulation	After ICH induction, mice were exposed to air, NBO, and 3ATA HBO for 60 min. The occludin, MMPs, and HIF-1α at 30 min, 60 min, and 120 min after treatment were compared among the three groups.	There was no significant difference between NBO and HBO in the early stage of oxygen therapy (within 30 min).	Inhibition of MMP-mediated proteolysis of blood–brain barrier components and hif-1a-induced upregulation of VEGF promotes occludin to protect BBB and reduce hemorrhagic edema.	(24)	
	ICH Rats	HIF/VEGF	Endothelial cell	Downregulation (early phase)				(24)	
	Cerebral ischemia–reperfusion injury Rats	AQP4	Neuron	Downregulation	Transient focal cerebral ischemia for 120 min followed by reperfusion for 48 h. Rats were exposed to 60 and 100% NBO or untreated for 48 h during reperfusion, respectively. AQP4, HIF-1α and BWC 24 h and 48 h were compared between them and sham operation group.	The content of AQP4 was significantly reduced in the treatment of 2–24 h after 100% NBO and 6 h after 60% NBO. 60 and 100% NBO treatment significantly reduced BWC at 48 h(The latter is not as effective as the former.)	NBO promotes AQP4 down-regulation, improves blood–brain barrier permeability, and reduces brain water content (BWC).	(29)	
	Cerebral ischemia–reperfusion injury Rats	HIF	Endothelial cell	Upregulation		The level of HIF increased significantly at 2 h and 12 h after 100% NBO and 2 h after 60% NBO. Both NBO treatment groups significantly reduced the volume of cerebral infarction at 48 h. (The latter is not as effective as the former.)	NBO up-regulates the level of HIF-1α, increases cerebral blood flow and reduces the volume of cerebral infarction.	(29)	
	Cerebral ischemia–reperfusion injury Rats	NHE1 (the membrane proteins Na/H exchanger 1)	Astrocyte	Downregulation		The level of NEH1 reduced significantly at 4 h after 100% NBO and 2 h after 60% NBO. (The latter is not as effective as the former.) The NIS of all NBO treatment groups decreased significantly at 24 h and 48 h.	NBO down-regulates NEH1 and improves NHISS	(29)	
	Health persons	HIF-1α	Endothelial cell	Upregulation	The level of HIF, NRF2, MMP9, GSH, and NF-κB protein in the nucleus of healthy subjects was measured at 30 min, 3 and 24 h after normoxia recovery after 1 h of 30%, 60%, 100%, and 140% oxygen therapy.	HIF-1α peaked at 3 h after 30% NBO.	Helps hypoxic tissue re-establish oxygen-supplying proteins.	(30)	
	Health persons	NRF2	Endothelial cell	Upregulation		At 100% NBO, NRF2 levels began to increase 30 min after returning to normoxia and returned to baseline at 24 h. In the 30 and 140% groups, NRF2 levels increased at 3 h after treatment and maintained for up to 24 h.	It can maintain intracellular redox homeostasis and reduce oxidative stress response. However, at 100 and 140% hyperoxia, the transition to oxidative stress occurred.	(30)	
	Health persons	MMP9	Neuron	Upregulation		The level of MMP-9 increased after 3 h of 30% NBO, and then returned to the basal level, which was consistent with the activation trend of HIF-1α. However, MMP-9 increased from 30 min under 100% NBO, and returned to the initial level at 3 and 24 h. The level of MMP-9 increased in a constant trend under 140% hyperoxia.	MMP-9 can be transformed to induce oxidative stress response under 100 and 140% hyperoxia.	(30)
	Health persons	NF-κB	Neuron	Upregulation		Activation began 30 min after 140% NBO.	The synergistic effect with HIF/NRF2 confers cell protection. When stress cannot be compensated, it can mediate programmed cell apoptosis.	(30)
	Health persons	GSH	Neuron	Upregulation		At 100% NBO, it increased significantly at 3 h after returning to normoxic state; the level of GSH in 140% O2 group was significantly increased at 3 and 24 h after treatment.	GSH is involved in cell protection, but it changes to oxidative stress under 100 and 140% hyperoxia.	(30)

This table lists the possible mechanisms and potential therapeutic effects of HBO and NBO with different concentrations and pressures in ICH and other diseases, in different experimental subjects such as humans and animals, and has reference significance for the selection of high-concentration oxygen therapy in ICH. HBO, Hyperbaric oxygen; NBO, Normobaric oxygen; ICH, Intracerebral hemorrhage; HIF-1α, Hypoxia-inducible factor1-alpha; MMP-9, pro-matrix metalloproteinase-9; TNF-α, Tumor necrosis factor-alpha; MACO, Middle cerebral artery occlusion; EPO, Erythropoietin; NF-kB, Nuclear factor kappa-B; CLI, Critical limb ischemic; Nrf2, Nuclear factor 2; NIHSS, National Institute of Health stroke scale; EPC, Endothelial progenitor cell; AQP4, Aquaporin4; GSH, Glutathione; BBB, Blood–Brain Barrier; NEH1, Na+/H+ exchanger 1; BWC, Brain water content.

### 2.1 Improve the ischemic and hypoxic state after ICH

Noemí et al. (31) conducted a longitudinal [18F]-fluorimidazole ([18F]-FMISO) PET/MRI study. They found that when the intracerebral hematoma volume peaked after ICH, the HIF-1α content in the damaged tissue was equal to that of [18F]-FMISO. The increased absorption of FMISO indicates a hypoperfusion state around the hematoma after ICH. Studies have found that HBO can supply oxygen to damaged areas of brain tissue, improve brain microcirculation, and promote angiogenesis in damaged brain tissue by promoting the regeneration of vascular endothelial cells (23). In an experiment to explore the effect of HBO on intracerebral blood vessels in rats after ICH, Zheng et al. found that on days 14 and 21, respectively, compared with the sham operation group and the ICH group, the HBO group had significantly increased HIF-1a expression and vascular endothelial growth factor (VEGF) positive microvessels. On days 14–28, the number of new blood vessels in the HBO group increased significantly. Combined with the rapid decline in behavioral scores in the HBO group over time, they believe HBO enables hematoma absorption by supplying oxygen and promoting the expression of HIF-1a and VEGF. Vascular endothelial cells proliferate, blood vessels regenerate and sprout, and cerebral blood flow significantly increases. Thus, oxygen and blood fully moisturize damaged brain tissue and neurons, ultimately improving brain microcirculation and nervous system damage after ICH. Some scholars have also discovered through ([18F]-FMISO) PET that early HBO can increase the glucose brain metabolism rate in ischemic tissue to a certain extent and effectively improve cerebral ischemia and hypoxia (26).

### 2.2 Reduce brain edema and damage to the blood–brain barrier

The severity of cerebral edema after ICH is closely related to the degree of brain damage. Therefore, reducing cerebral edema is an entry point for ICH treatment, and HBO has been shown to reduce cerebral edema in previous studies (32). In an experimental study (24), Zhou et al. found that after ICH induction, the aggravation of vasogenic edema accompanied an increase in barrier permeability, and the levels of pro-matrix metalloproteinase-9 (proMMP-9), matrix metalloproteinase-9 (MMP-9), and occludin in the bleeding hemisphere were significantly increased. Early initiation of HBO could reduce the levels of proMMP-9 expression, thereby avoiding the activation of MMP-9 and the destruction of occludin. Simultaneously, edema and destruction of the blood–brain barrier were significantly alleviated. These results demonstrate that HBO reduces brain edema and blood–brain barrier permeability after ICH by inhibiting the activation of MMP-9. MMP-9 belongs to the matrix metalloproteinase family. Its activation can degrade blood–brain barrier tight junction proteins, increase blood–brain barrier permeability, and promote brain edema. Tight junction proteins, including occludin, claudins, and zonula occludins (ZO-1, ZO-2, ZO-3), are essential for maintaining the stability of endothelial cells in the blood–brain barrier (33).

### 2.3 Constrict blood vessels

In a study by Yamamoto et al. (21), it was found that when exposed to HBO for a short period, endothelial cells produced the vasodilators nitric oxide (NO) and superoxide (O2-), and their combined product, peroxynitrite (ONOO-) can cause vasoconstriction and decrease blood flow, which appears to contradict the treatment needs of ICH. Interestingly, over a longer period, HBO can fully compensate for this reduction in CBF, resulting in an overall improvement in microinflammation, circulation, blood flow, and oxygen tension. From another perspective, HBO-induced vasoconstriction can reduce the extravasation of tissue fluid and cerebral blood volume, thereby reducing intracranial pressure. A reduction in intracranial pressure is also beneficial for alleviating brain swelling, ischemia, and oxygen deficiency (19).

### 2.4 Maintain the body’s oxidation and antioxidant balance and improve antioxidant activity

After ICH, a large amount of heme is released from damaged red blood cells and infiltrates the surrounding hematoma of the injured brain tissue. After metabolism, the heme releases ferric iron. Iron overload induces excessive reactive oxygen species (ROS) production and oxidative stress. These effects may be one of the causes of increased lipid peroxidation observed after ICH (34). The same finding was reported in a previous clinical study. Compared to healthy subjects, the expression of low-density lipoprotein in the plasma of patients with acute stroke was significantly increased, indicating that the lipid peroxidation level of the latter was significantly higher than that of the former (35). It is clinically believed that the time of HBO treatment is controlled within 1 h and the pressure is controlled between 2-3ATA, which can reduce the probability of hyperoxia damage to the body (36). Oxidative stress refers to the body’s excessive production of oxygen free radicals under exogenous stimulation, leading to an imbalance in the body’s oxidation-antioxidation system. It can destroy normal cells, lipids, and proteins and damage the central nervous system (37). Therefore, active measures should be taken to fight against it. Breitenbach et al. believe that HBO can maintain the body’s oxidative and antioxidant balance as long as it is within the therapeutic and approved range. Within this range, the body’s spontaneous anti-oxidant mechanism is sufficient to combat the oxidative stress damage caused by HBO. HBO can also evoke antioxidant enzyme activity through the nuclear factor 2 (Nrf2) pathway, effectively reducing ROS levels. Furthermore, long-term HBO treatment can reduce mitophagy-induced apoptosis by enhancing mitochondrial activity (36). In other diseases, such as fibromyalgia, HBO has been shown to reduce lipid peroxidation levels and alleviate oxidative stress (38).

### 2.5 Reduce neuroinflammation and neuronal degeneration caused by ICH

Clinical studies show (22) that HBO has a long-term neurological improvement effect in patients with diabetes and hemorrhagic stroke, Qian Xu et al. randomly divided 79 eligible patients into two groups, 31 patients in the HBO group received 2.5ATA for 60 min, and 48 patients in the NBO group received 1,5ATA for 60 min. There was no significant difference in the outcomes between the two groups 1 month after the onset of the disease, but 6 months after the onset of the disease, compared with the NBO group, the efficacy of the HBO group was significantly improved, especially in the two scales of Modified Rankin score and National Institute of Health Stroke Scale (NIHSS), they believed HBO may have functions such as increasing oxygenation, reducing cerebral edema, and regulating glial cell metabolism. Microglia are the guards of the brain. They are the first to activate the immune defense line after ICH and play an important role in clearing hematomas, engulfing damaged cells, and reducing inflammation (39). Lim-ing Yang et al. found through Iba1-DAPI double staining that compared with the ICH group, the microglia around the hematoma in the hyperbaric oxygen preconditioning (HBOP) group showed a significant downward trend at 12, 24, and 72 h. Fluoro-Jade C detection revealed that the hematoma and peripheral neuron degeneration in the HBOP group were also significantly reduced compared to that in the ICH group. Based on the neurobehavioral scores of the three groups of mice, they believed that HBOP reduced neuroinflammation by regulating the expression of microglia after ICH (25). When a patient undergoes HBOP, the partial pressure of oxygen in the blood remains high for some time. Therefore, clinical research has been conducted on the effects of administering HBO before ICH. This treatment method, called hyperbaric oxygen preconditioning (HBOP), also protects fragile nerves in ICH and has achieved clinical success (40). Some studies have also found that HBO has significantly more vascular endothelial cells and microvascular structures on 14d ~ 28d than the ICH group on 14d ~ 28d, and the behavioral scores of mice in the HBO group decreased significantly over time. Therefore, they believed that HBO could upregulate the expression levels of HIF-1α, VEGF, and other factors can promote vascular endothelial cell proliferation, angiogenesis, and hematoma absorption, alleviating damaged brain tissue’s ischemic and hypoxic state and improving nerve cell function and behavioral defects (23).

### 2.6 The possible effects of different ICH models on oxygen therapy

There are differing opinions on the optimal pressure and exposure time for HBO in experimental studies. These differences may be because of the different selection of experimental subjects and methods used to induce ICH models. Based on the treatment methods described in the literature, we believe that the two most commonly used animal models of ICH are injection of collagenase and infusion of autologous blood. Although both are not identical to the pathophysiology of clinical ICH, they provide a molecular basis. The former may be due to the fact that collagenase can degrade the basal interstitium, basement membrane, and blood leakage of blood vessels, leading to inflammation, edema, and destruction of the blood–brain barrier, which could be more dangerous than the clinical manifestations (6, 41, 42).

Meanwhile, the latter better imitates the blood mass effect, allowing for better efficacy in experimental studies of HBO treatment of ICH. In animal experiments, we also found that the therapeutic effect of rabbits may be better than that of mice (6, 41, 42). However, the results of animal experiments cannot be completely copied into clinical practice (1). Additional clinical studies are required to support this argument.

### 2.7 Hyperbaric oxygen precautions

Based on current experimental research, long-term exposure to HBO with inappropriate pressure (pressure between 4 and 5 ATA, lasting more than 2 h) can easily induce central nervous system poisonings such as epilepsy, retinal damage, and pulmonary toxicity. This effect may be because the body produces excessive oxygen-free radicals, which leads to the exhaustion of antioxidants, causing oxidative stress damage, cell necrosis, and apoptosis (43–45). However, these side effects can be avoided by choosing an appropriate pressure and exposure time (44). In one study, it was considered safe to start the air brake every 20 to 30 min after exposure to 2.4 ATA for 60 to 90 min (46). After analyzing 22 patients with acute stroke who received HBO, McCormick et al. (47) believed that the most effective time window for HBO is within 3 h after ICH, and the earlier oxygen is administered, the better the effect. Their findings indicate that there is a time window for hyperbaric oxygen therapy. Further research is required to explore and standardize the safety of HBO. In addition, given the tightness and narrowness of the HBO treatment chamber, ICH patients need to be treated in the chamber alone for up to 2 h, which is very difficult for some patients with low compliance, especially those with severe conditions and high-risk factors for epilepsy. If there is a sudden attack during the treatment, the hyperbaric oxygen chamber cannot be abruptly stopped like MRI, because it involves the process of decompression, which is one of the limitations of HBO treatment (48). In addition, the paralysis rate of ICH is high, and most patients still have sequelae within a short period, requiring medical staff and family members to push them to the HBO treatment cabin, which reflects the inconvenience of HBO.

## 3 Normal-pressure high-concentration oxygen

NBO is a treatment method that provides high-concentration oxygen through a mask or nasal cannula at sea level (1ATA) to increase the oxygen partial pressure. The oxygen concentration fraction was greater than 30% (10). NBO has low cost, high portability and safety, and no restrictions on its place of use. It is suitable for the use of ambulances when transporting patients with sudden ICH. Zhou et al. (24) found no difference in efficacy between HBO and NBO within 30 min of ICH, illustrating the effectiveness of the early adoption of NBO after ICH. NBO is also suitable for bedridden patients with limited mobility or home-based oxygen therapy after discharge. Its treatment mechanism is similar to that of HBO and is clinically effective in improving the damage and prognosis of ICH. Therefore, we recommend expanding the therapeutic applications of NBO after ICH.

### 3.1 Improve the ischemic and hypoxic state after ICH

NBO can relieve ischemia and hypoxia following ICH by improving oxygenation and reducing cerebral edema damage (49). You et al. (50) found through animal experiments that compared with the control group, the NBO group could significantly reduce the water content of damaged brain tissue, downregulate the initial expression of HIF-1α and VEGF, reduce cell apoptosis, and improve damaged nerve function, especially oxygen. The NBO group with a concentration of 90% showed the best efficacy. As mentioned earlier, HIF-1α and VEGF also benefit angiogenesis during brief hypoxia, thereby increasing cerebral blood flow and improving cerebral oxygenation and microcirculation (23).

### 3.2 Reduce brain edema and damage to the blood–brain barrier

In addition to improving the water content of tissue around hematoma by regulating the expression of HIF-1α and VEGF, NBO can also reduce brain edema by down-regulating the expression of aquaporin4 (AQP4). An animal experiment found that the AQP4 content of mice in the NBO group treated with 60 and 100% oxygen was significantly lower than that in the sham surgery group (29). AQP4 is a water-permeable channel protein, and its knockout can greatly reduce brain edema (51). Low AQP4 expression can indirectly alleviate secondary vasogenic edema by reducing initial cytotoxic edema after ICH, thereby improving blood–brain barrier permeability (52). NBO can also protect the blood–brain barrier by inhibiting the activity of MMP-9 to alleviate the degradation of occludin (53). This protective effect was also demonstrated in other studies (54).

### 3.3 The protective effect of the normobaric oxygen paradox

Clinically, intermittent NBO is administered to patients with ICH based on the paradox of normal-pressure oxygen, similar to HBO. The normobaric oxygen paradox refers to the relative hypoxic response of the body when hyperoxia fades and returns to normal oxygen levels. Hypoxia can trigger cellular cascade reactions and stimulate tissue regeneration without adverse effects (46). After exposure to 30 and 100% NBO for 60 min, the former showed hypoxic stress, whereas the latter showed oxidative stress (55). Under 30% NBO, HIF-1α can be activated by hypoxia stress; however, at 100% NBO, the protective effect of atmospheric oxygen paradox decreased. The activation of Nrf2 and NF-κB and the production of glutathione (GSH) reflected the enhancement of oxidative stress (30). We can use the normobaric oxygen paradox to promote the efficacy of NBO after ICH, but we should also pay attention to avoiding the side effects caused by excessive oxygen concentration.

### 3.4 Protect neurological function

A clinical study showed that NBO protects neuronal function and improves oxygenation by reducing lactic acid (Lac) and N-acetyl-aspartate (NAA) (56). Henninger et al. (57) found that NBO could reduce cell apoptosis around the ischemic penumbra, provide lasting nerve protection, and prolong the reperfusion time window. NBO has been proven to have excellent efficacy in early stroke. However, some studies have found that NBO combined with n-acetylcysteine (NAC) can exert a more powerful neuroprotective effect, which is similar to HIF-1α, VEGF, and poly ADP-ribose polymerase (PAPR-1), the degradation of tight junction proteins is closely related (58). In addition, NBO can enhance the neuroprotective effects of melatonin (59), minocycline (60), ethanol (61), and other drugs.

### 3.5 NBO treatment considerations

In addition to the influence of the animal model induction method mentioned above, NBO treatment should also focus on exposure time and concentration. Zhou et al. (24) found that oxygen therapy was effective in treating ICH only if administered within 30 min of ICH. However, this experiment has some limitations. Different oxygen concentrations and pressures produced different effects. No hierarchical surveys were conducted in this experiment. Long-term exposure to inappropriate concentrations of NBO produces excessive oxygen free radicals, increases lipid peroxidation levels, and damages mitochondrial function. Oxygen toxicity can ultimately lead to adverse reactions such as hyperoxic acute lung injury (62). Therefore, NBO with an oxygen concentration of 41%–90% provides intermittent treatment to avoid adverse reactions and regularly check blood pondus hydrogenii (PH) (63). In addition to vitamins C and E, which can reduce oxidative damage (63), curcumin (64), its analogs (65), and resveratrol (66) can also reduce lung damage, which can be considered if necessary (67). In addition, it should be noted that hyperoxia therapy is not recommended for severely ill patients (67). It should also be noted that NBO is mainly administered through tracheal intubation, and long-term tracheal intubation will inevitably cause damage to the patient’s throat (68) and even bring risks to patients with severe laryngeal edema (69). This suggests that we should develop NBO treatment time according to the patient’s condition, or alternate NBO and HBO treatment as a new direction for future exploration.

## 4 Other possible effective molecular mechanisms of high-concentration oxygen therapy

### 4.1 HIF-1α/BNIP3/Beclin1 pathway promotes mitophagy

Under normal physiological conditions, HIF-1α is almost not expressed. However, when the tissue is hypoxic, the E3 ubiquitin ligase is recruited by the tumor suppressor protein von hippel–lindau (VHL), ubiquitination of this pathway is inhibited (70), and asparagine hydroxylase factor (FIH) binds to the hypoxia response element (HRES) in the nucleus and increases HIF transcriptional activity (71). The body is in a state of hypoxia after ICH, so HIF-1α shows a high expression level (72); at this time, HIF-1α can up-regulate the levels of pro-apoptotic gene BNIP3 and Beclin1 through the HIF-1α/BNIP3/Beclin1 pathway and promote mitophagy (73). Ostrowski et al. (74) exposed mice 1 h after subarachnoid hemorrhage (SAH) to 2.8 ATA HBO for 2 h. After 24 h, HIF-1α was significantly increased in the untreated SAH group and significantly increased in the HBO-treated group; at the same time, the BNIP3 level in the HBO group decreased to the level of the sham operation group. Although the brain needs BNIP3 to remove necrotic cells and promote apoptosis after ICH, mitophagy is a double-edged sword beneficial for clearing excess ROS, damaging mitochondria, and protecting neurological function after ICH (75, 76). However, excessive autophagy activates NADPH oxidase, producing excessive ROS, engulfing normal cells, and aggravating damage (77). Therefore, after ICH, timely administration of medical oxygen is essential, and monitoring the pressure, concentration, and exposure time to medical oxygen is crucial to avoid adverse injuries.

### 4.2 EPO/JAK/STAT pathway antagonizes neuronal apoptosis

In brain tissue with focal ischemia, HIF transcriptional activity is enhanced after HBO pretreatment, the expression of its downstream target gene EPO is increased, and the two are regulated positively (28). In addition, the neuroprotective functions of EPO have been widely reported in both clinical and experimental studies. Fernando et al. found that when rats with middle cerebral artery occlusion (MCAO) received exogenous EPO, the apoptosis marker anti-cleaved caspase-3 (CC3) decreased significantly, and the cell proliferation marker Ki67 increased more than in the control group. In clinical trials on neonatal stroke, EPO was found to mobilize the proliferative activity of neurons and oligodendrocytes in the early stages of stroke, inhibit astrocyte generation, and play a neuro-protective role (78). Xiong et al. (79) also found that early EPO treatment after traumatic brain injury (TBI) can significantly restore sensorimotor and spatial learning functions. EPO activates a series of pathways by binding to the homodimer EPOR, leading to autophosphorylation of Janus kinase 2 (JAK2), including signal transducer and activator of transcription 5 (STAT-5), phosphatidylinositol 3-kinase (PI3K)/AKT and SHC (SH2 adapter protein C)/mitogen-activated protein kinase (MAPK) (80), thereby playing an anti-apoptotic and neuroprotective role (78, 81). Among them, the JAK/STAT pathway is the most commonly used pathway to provide EPO-induced neuroprotective effects. Phosphorylated JAK2 is involved in the phosphorylation of tyrosine residues in EPOR cytoplasm; these residues can bind to the SH2 domain of STAT5; when brain trauma occurs, STAT5 activity can be inhibited by negative feedback factors such as SOCS-3 activated by proinflammatory cytokines, thereby preventing EPO’s intracellular signal transduction and weakening EPO’s neuroprotective effect (82); this pathway can be blocked by JAK2 inhibitors (83). Zhang Feng et al. (83) found that the phosphorylation of STAT5 was enhanced under the induction of EPO, and the expression of its downstream target genes B-cell lymphoma-extra-large (BCL-xL) and X-linked inhibitor of apoptosis (XIAP) also increased, these two target genes are one of the genes that inhibit the mitochondrial apoptosis pathway.

### 4.3 VEGF/PI3K/AKT and ERK promote vascular remodeling

HIF regulates multiple target genes, including vascular endothelial growth factor (VEGF). During hypoxia, HIF transcriptional activity increases, and VEGF expression is upregulated. VEGF plays a major role in early embryonic development and angiogenesis (84). It can promote angiogenesis and endothelial cell migration and proliferation (85). Under hypoxia, the transcriptional activity of HIF increased and the expression of VEGF was up-regulated. Repeated intermittent hyperoxia treatment after ICH can increase the expression of HIF, VEGF, and endothelial progenitor cells (EPC), increase the number of new blood vessels and cerebral blood flow in the damaged brain tissue, and improve the neuroinflammatory response and damaged cells due to rich blood supply and oxygen supply (23, 27). Among them, VEGF can promote the extracellular signal-regulated kinase (ERK) and PI3k/AKT pathways by activating vascular endothelial growth factor receptor 2 (VEGF-R2) (86). Jing et al. found that when the injured blood vessels were exposed to 30% NBO, the levels of ERK and protein kinase B (AKT) were significantly lower than those in the control group receiving 21% oxygen, indicating that hyperoxia inhibited the phosphorylation of ERK and PI3K; at the same time, the proliferation of smooth muscle cells (SMC) in injured vascular smooth muscle cells in the 30% NBO group was significantly inhibited, which was similar to the changes of ERK and AKT levels in the same group (87). SMC is the main cell type in vascular walls, being an important cell type involved in vascular remodeling and blood distribution control (88), which has positive significance for the prognosis of ICH. Notably, EPO can up-regulate the production of VEGF through the PI3K/AKT and ERK1/2 pathways and promote the secretion of VEGF-R2 by neural progenitor cells, which can synergistically promote neurovascular remodeling and neurobehavioral improvement (89, 90).

### 4.4 HIF-1α/COX pathway and other pathways improve oxygenation

**Figure 2:**
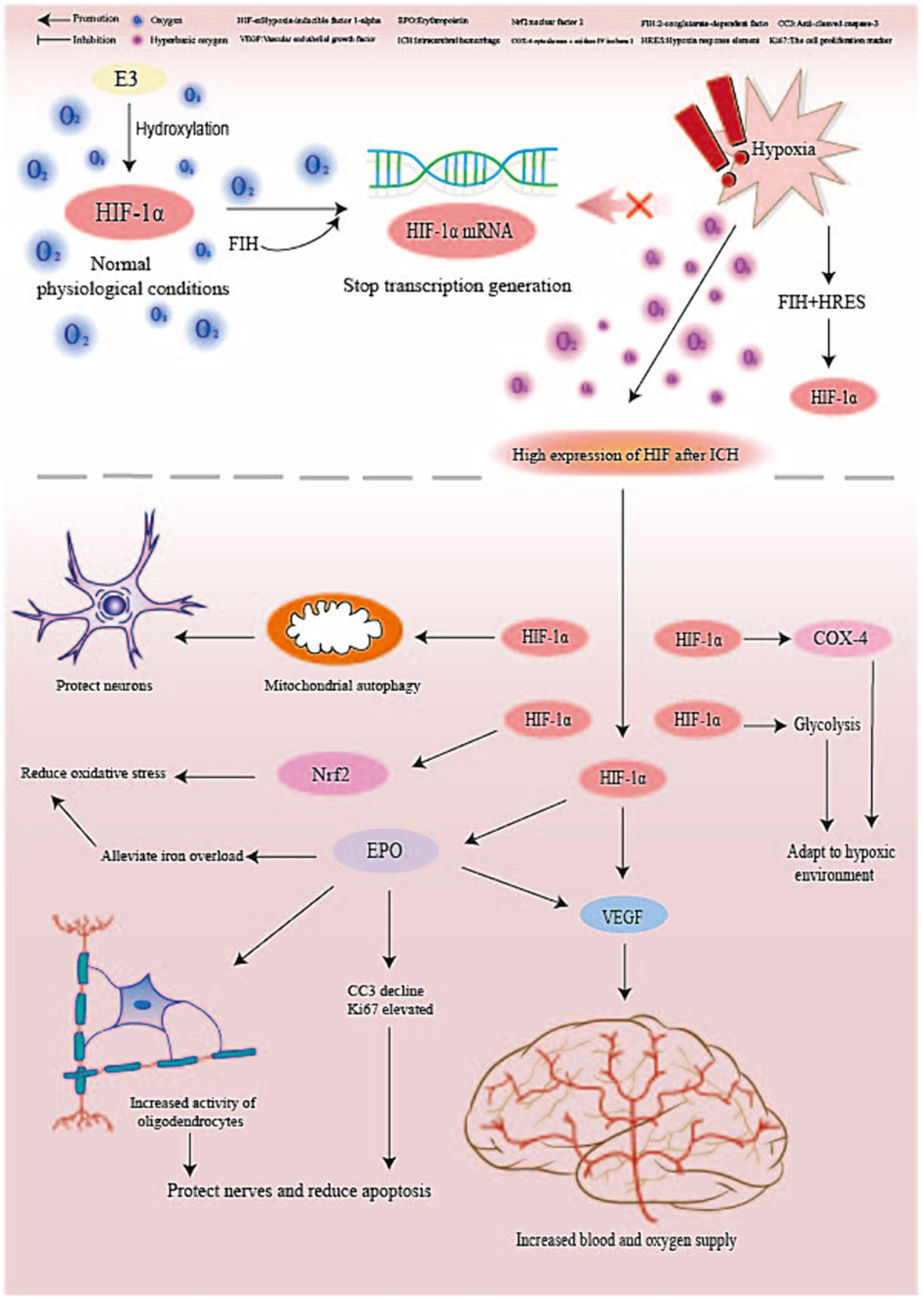
Molecular pathways that may play a role in high-concentration oxygen therapy.

HIF-1α can improve the oxygen metabolism and mitochondrial function of hypoxic cells after ICH and promote tissue to overcome pathological hypoxic injury by regulating oxygenation (71, 91). When the tissue is anoxic, the mitochondrial respiratory function is impaired, resulting in the aggregation of the reducing equivalent at cytochrome c oxidase (COX) rather than oxygen, so the electrons prematurely react with the molecular oxygen in complexes 1 and 3 to generate superoxide (O2-) (92). O2- is a kind of ROS that is closely related to inflammatory reactions and is not conducive to the prognosis of ICH (93). However, HIF-1α expression increases under hypoxia, and COX4-1 in normoxia is degraded by mitochondrial protease LON, which activates COX4-2 production. The latter can increase the levels of H_2_O_2_ and ATP and improve tissue oxygenation and electron respiratory chain activity (94). In addition, HIF-1α can enhance glycolysis and reduce mitochondrial oxygen consumption to adapt to a hypoxic environment and avoid excessive ROS production (95). EPO regulates the production of erythroferrone (ERFE) by erythroblasts, inhibits hepcidin activity, enhances iron absorption and storage, promotes hematopoietic function (96), and relieves iron overload (96). Thus, the oxidative stress caused by iron overload can be improved (34). (The details are shown in Figure 2.)

## 5 The effective treatment range of high-concentration oxygen therapy in ICH

In order to avoid oxygen toxicity to the greatest extent and improve efficacy, HBO exposure for 1 h under 2-3ATA is usually used clinically (36). Some scholars also believe that the use of 2.4 ATA HBO treatment for 60–90 min and activation of the air brake program every 20–30 min can bring positive results (45). Lan et al. (39) suggested that a single exposure to 2.5 ATA HBO for 1 h, once a day for 30 days after ICH can significantly improve NIHSS. Zhou et al. (24) found that giving 3ATA HBO 1 h at the very early stage (within 30 min) after ICH could also improve cerebral microcirculation and reduce cerebral edema. However, Qin et al. (97) found that under the same 3 ATA HBO, a single exposure of 1 h can significantly reduce the volume of PHE at 24 h, while the treatment of 3 times within 24 and 1 h each time cannot reduce the volume of PHE at 72 h, and even increase the toxicity of iron. This suggests that we should not only pay attention to the pressure range and interval time of HBO, but also the number of treatments.

For the safe concentration of NBO, the clinical recommendation is 41%–90% (63). Fratantonio et al. (30) though 100% of NBO can also promote the increase of HIF-1α, the excessive concentration will promote the oxidative stress response, which will make the body spend more time to restore balance. Zhou et al. (24) found that there was no significant difference between the effect of NBO treatment and HBO treatment in the very early stage (within 30 min) after ICH.

## 6 Summary

This article elaborates on the hypoxic pathological process of ICH and the molecular mechanisms by which HBO and NBO may be effective against ICH. Combined with the latest research, we draw the following conclusions. (i) High-concentration oxygen therapy can damage and protect the brain after ICH and plays an important and positive role in the outcome; (ii) Understanding the duration, concentration, pressure, and treatment interval of patients with ICH and exposure to hyperoxia can effectively avoid hyperoxia toxicity. (iii) There may be a specific time window for hyperoxia therapy following ICH. Hyperoxic therapy after ICH can reduce cerebral edema and oxidative stress, reduce ICP, increase CBF and cerebral oxygenation, protect neurological function, and improve the prognosis of patients with ICH. We recommend intermittent administration of high-concentration oxygen therapy to avoid oxygen toxicity and improve efficacy. We believe that the air brake is activated every 20–30 min within 60–90 min of HBO at 2.4 ATA (46) or NBO with an oxygen concentration of 41%–90% under normal pressure (63). There was no significant difference in the efficacy of NBO and HBO in the very early period after ICH, and the earlier oxygen was administered, the better the efficacy. When giving patients high-concentration oxygen therapy for the first time after the onset of the disease, NBO is the preferred ICH treatment. This article summarizes three directions that need to be studied in the future: (i) Defining the interval duration, concentration, and pressure of hyperoxic therapy after ICH to maximize efficacy. (ii) Is there a more precise time window for hyperoxia treatment after ICH onset? and (iii) Is there an outbreak of oxygen toxicity during long-term treatment?

Regarding long-term effects, HBO appears more effective than NBO regarding brain metabolism (98). Perhaps combined therapy can be used to maximize the advantages of hyperoxia therapy. Studies have found that, in TBI, the efficacy of HBO + NBO as a single treatment is more significant than that of the two treatments alone. Oxygen toxicity markers were significantly reduced, cerebral microcirculation and mitochondrial function were improved, ICP and mortality were reduced, and prognosis was better (99). These findings serve as a significant reference point for the treatment of ICH. In the future, we will explore the mechanism of combined HBO + NBO therapy in ICH and how to select a treatment plan to achieve the best effect of synergistic therapy.

## Author contributions

HZ: Conceptualization, Data curation, Formal analysis, Writing – original draft. DZ: Conceptualization, Data curation, Formal analysis, Writing – review & editing. XY: Conceptualization, Data curation, Formal analysis, Writing – review & editing. WZ: Conceptualization, Data curation, Formal analysis, Writing – review & editing. MW: Conceptualization, Data curation, Formal analysis, Writing – review & editing. ZC: Conceptualization, Data curation, Formal analysis, Writing – review & editing.

## Figures and Tables

**Table:** 

HIF-1α	Hypoxia-inducible factor 1-alpha
ICP	Increase intracranial pressure
CPP	Cerebral perfusion pressure
PHE	perihematomal edema
NBO	Normobaric oxygen
proMMP-9	pro-matrix metalloproteinase-9
ZO	Zonula occludins
ROS	Reactive oxygen species
HBOP	hyperbaric oxygen preconditioning
NF-κB	Nuclear factor kappa-B
NAC	N-acetylcysteine
VHL	Von Hippel–Lindau
PH	Pondus Hydrogenii
COX	cytochrome c oxidase
SAH	Subarachnoid hemorrhage
CC3	Marker anti-cleaved caspase-3;
EPOR	Erythropoietin receptor
PI3K	Phosphatidylinositol 3-kinase;
TBI	Traumatic brain injury
EPO	Erythropoietin
PARs	protease-activated receptors
CVR	cerebral vascular resistance
JVR	jugular venous reflux
[18F]-FMISO	[18F]-fluorimidazole
MMP-9	Matrix metalloproteinase-9
O2-	superoxide
Nrf2	Nuclear factor 2
AQP4	Aquaporin4
Lac	Lactic acid
PAPR-1	Poly ADP-ribose polymerase
FIH	Asparagine hydroxylase factor
BNIP3	Pro-apoptotic gene
EPC	Endothelial cells
ERK	Extracellular signal-regulated kinase
MCAO	Middle cerebral artery occlusion
JAK2	Janus kinase 2
SHC	SH2 adapter protein C
AKT	protein kinase B
ICH	Intracerebral hemorrhage
TNF	Tumor necrosis factor
CBF	cerebral blood flow
HBO	Hyperbaric oxygen
VEGF	Vascular endothelial growth factor
NO	Nitric oxide
ONOO-	peroxynitrite
NHISS	National Institute of Health Stroke Scale
GSH	glutathione
NAA	N-acetyl-aspartate;
BCL-xL	B-cell lymphoma-extra-large
HRES	Hypoxia response element
XIAP	X-linked inhibitor of apoptosis
SMC	Smooth muscle cell
VEGF-R2	Vascular endothelial growth factor receptor 2
Ki67	Cell proliferation marker;
STAT-5	Transcription 5
MAPK	Mitogen-activated protein kinase
COX	Cytochrome c oxidase
